# RESILIENCE: A “CURE” FOR OR A “RESPONSE” TO AGING?

**DOI:** 10.1093/geroni/igae098.0924

**Published:** 2024-12-31

**Authors:** Chao Fang, Alastair Comery, J D Carpentieri

**Affiliations:** University of Bath, Liverpool, England, United Kingdom; University of Bath, Bath, England, United Kingdom; University College London, London, England, United Kingdom

## Abstract

Resilience is often perceived as an intrinsic ability to adapt to challenges. Grounded in the human psyche, research commonly depicts it as a self-defence mechanism, enabling people to navigate and overcome adversity. Sociological thinking questions this view, suggesting that resilience should not merely involve adaptation but also foster positive change in challenging situations. This perspective criticises resilience for potentially reinforcing a neoliberal ideology, shifting welfare responsibility from the state to individuals. Using longitudinal qualitative data from people over 50 with Long COVID in the UK, we argue that resilience is an embodied construction rooted in life narratives and framed by social dynamics. Examining three in-depth case studies with distinctive resilience trajectories—decline, improvement, and persistence—between 2021-23, we explore resilience amidst the challenges of Long COVID and ageing. Inherent resilience is not always stable or positive; it operates like the immune system, resisting disruptions amid the challenges of ageing and the struggles of navigating everyday life. By presenting data from people approaching the third age (early 50s) to those in their 60s and 70s, we argue that resilience is rooted in narrative identity, maintaining consistency in life plots. It involves drawing on past experiences, developing a flexible future outlook, and living for the present while navigating Long COVID. Our analysis uncovers the existential dimension of resilience in old age, a constant questioning of one’s existence through their body and health. It highlights how social support can alleviate such challenges, contributing to our understanding and support for the complex nature of ageing.

